# Supplementary material for: Sample-to-answer platform for the clinical evaluation of COVID-19 using a deep learning-assisted smartphone-based assay
Source: Nat Commun. 2023 Apr 24;14:2361. doi: 10.1038/s41467-023-38104-5 (PMC10124933; doi:10.1038/s41467-023-38104-5)
Supplement: Supplementary file 1 — Supplementary Information File [file 41467_2023_38104_MOESM1_ESM.pdf]

# Supplementary Information

# Sample-to-answer Platform for the Clinical Evaluation of COVID-19 using a Deep Learning-assisted Smartphone-based Assay

Seungmin Lee[1,2,*], Sunmok Kim[1,*], Dae Sung Yoon[2,3,4*], Jeong Soo Park[1], Hyowon Woo[1], Dongho Lee[5], Sung-Yeon Cho[6,7], Chulmin Park[6], Yong Kyoung Yoo[8,†], Ki-Baek Lee[1,†], and Jeong Hoon Lee[1,†]

[1] *Department of Electrical Engineering, Kwangwoon University, 20 Kwangwoon-ro, Nowon, Seoul 01897, Republic of Korea*

[2] *School of Biomedical Engineering, Korea University, 145 Anam-ro, Seongbuk, Seoul 02841, Republic of Korea*

[3] *Interdisciplinary Program in Precision Public Health, Korea University, Seoul 02841, South Korea*

[4] *Astrion Inc, Seoul 02841, South Korea*

[5] *CALTH Inc., Changeop-ro 54, Seongnam, Gyeonggi 13449, Republic of Korea*

[6] *Vaccine Bio Research Institute, College of Medicine, The Catholic University of Korea, Seoul, Republic of Korea*

[7] *Division of Infectious Diseases, Department of Internal Medicine, College of Medicine, The Catholic University of Korea, Seoul, Republic of Korea*

[8] *Department of Electronic Engineering, Catholic Kwandong University, 24, Beomil-ro 579 beon-gil, Gangneung-si, Gangwon-do 25601, Republic of Korea*

* These authors contributed equally.

† Corresponding authors: yongkyoung0108@cku.ac.kr, kblee@kw.ac.kr, jhlee@kw.ac.kr

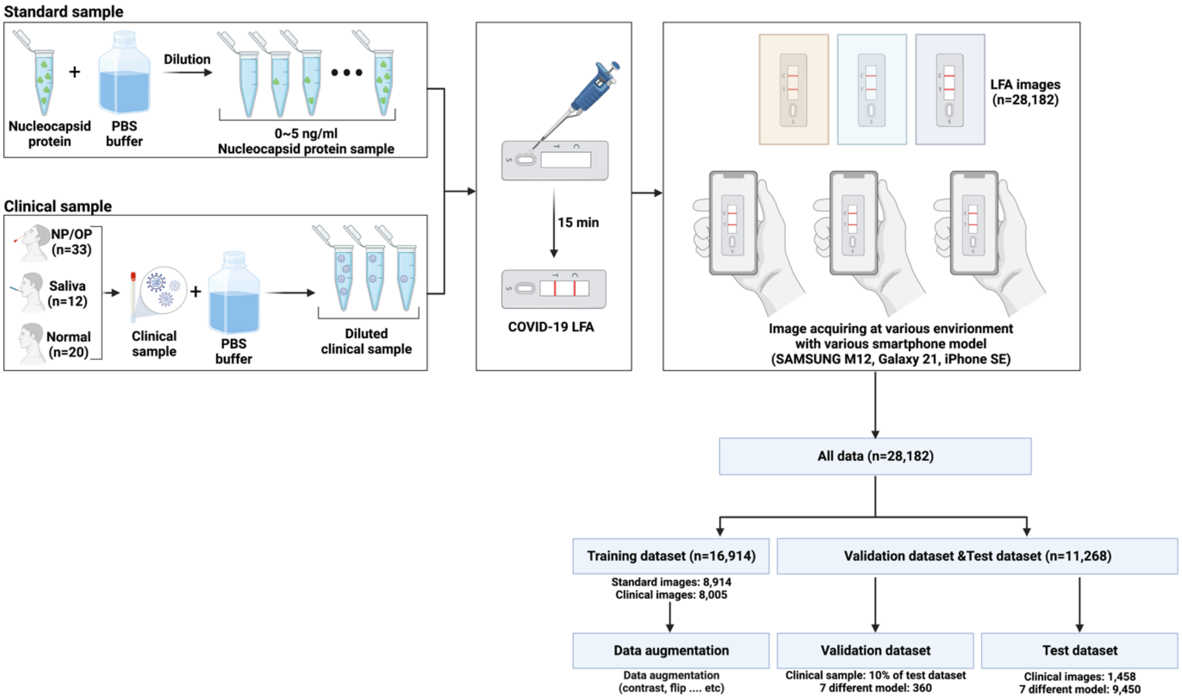


**Supplementary Fig. 1. Data preparation for SMART$^{AI}$-LFA.**

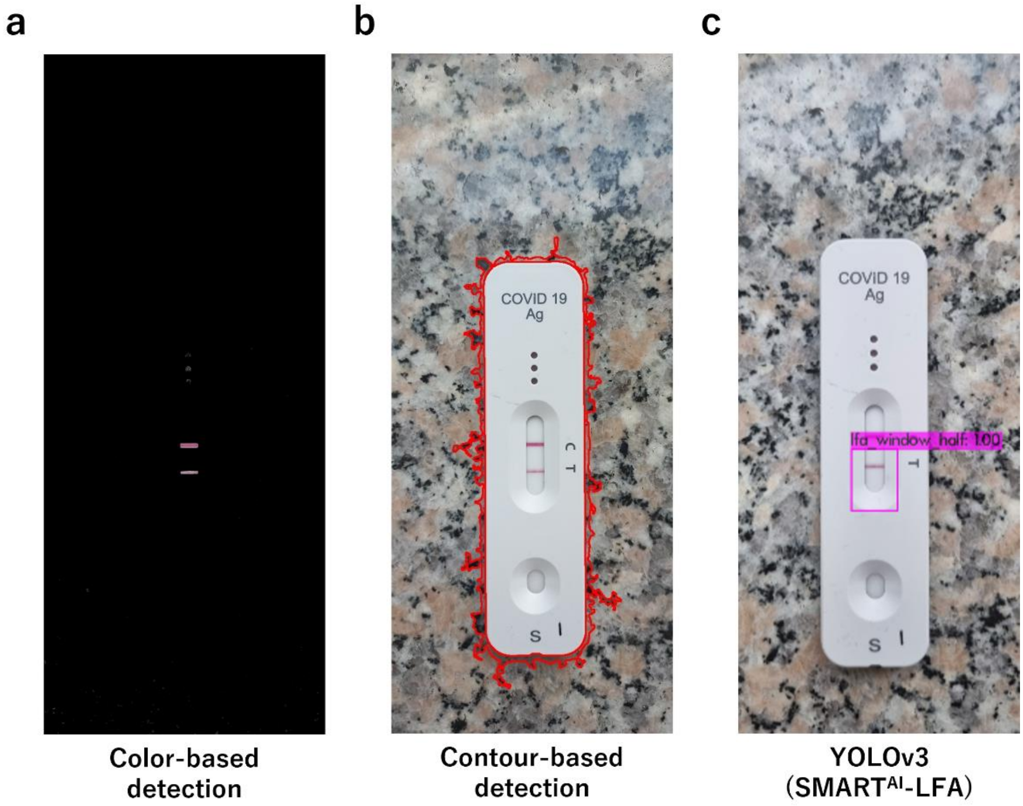


**Supplementary Fig. 2. Object finding models; two commercially available models and our SMART$^{AI}$-LFA model.** Two available models of (a) color-based detection, which detect red in the LFA kit and inevitably limit performance in the low test line signals, and (b) contour-based detection, which is limited by the background color or patterns of the image. (c) The YOLOv3 model for automatic object finding shows enhanced accuracy without the effects of the background and test line signals.

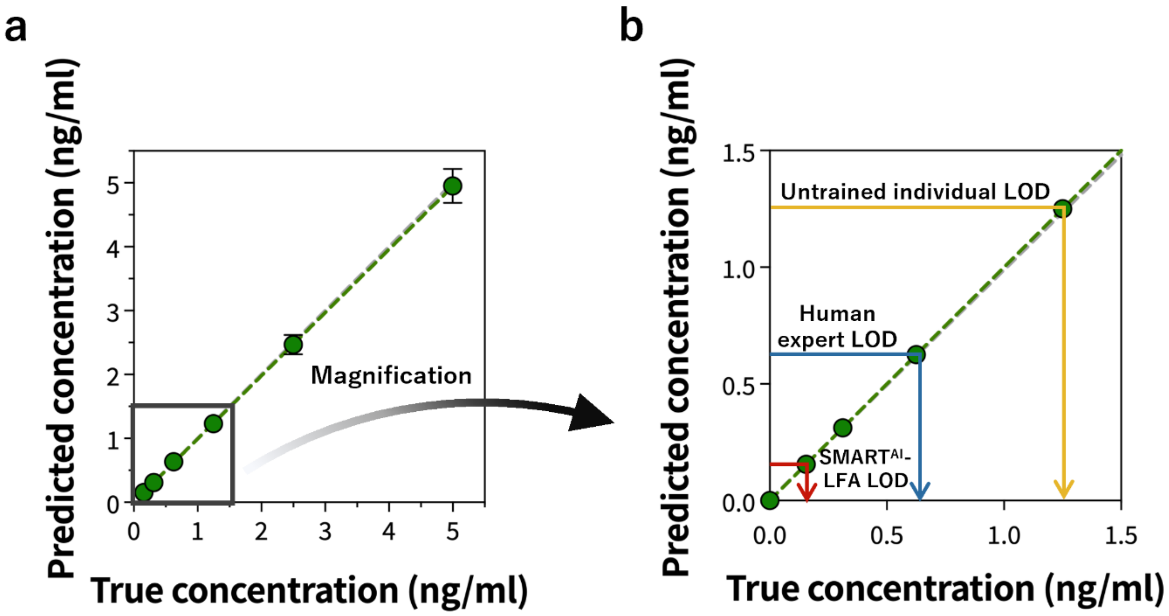


**Supplementary Fig. 3. Concentration prediction of untrained individuals, human experts, and SMART$^{AI}$-LFA. (**a) The plot of true concentration versus predicted concentration (n=70) showed the $R^2$ value reaching 0.99. (b) A magnified view revealed that the LODs were 1.25, 0.625, and 0.156 ng/ml for the untrained individuals, human experts, and SMART$^{AI}$-LFA, respectively. Error bars represent standard deviation from the mean.

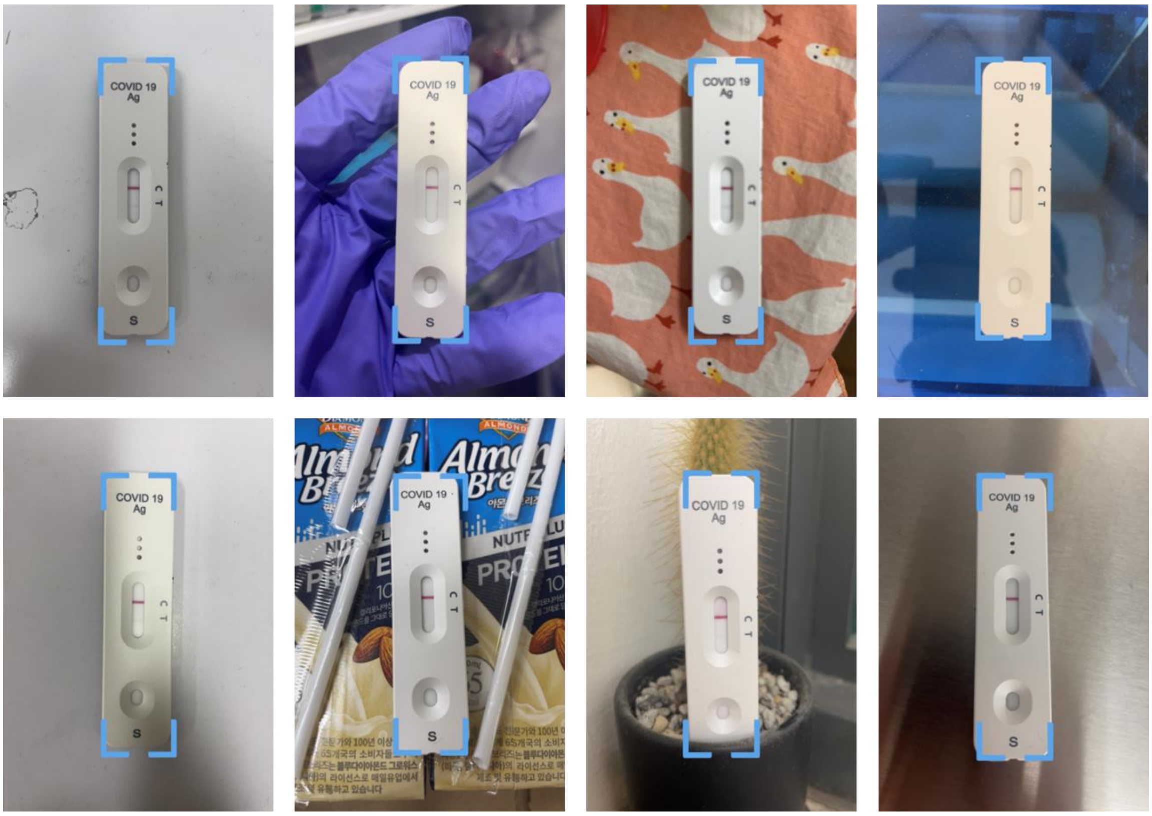


**Supplementary Fig. 4.** Examples of various surroundings such as indoors/outdoors, lighting conditions, and shade/sunlight with various backgrounds for smartphone app-based diagnostics.

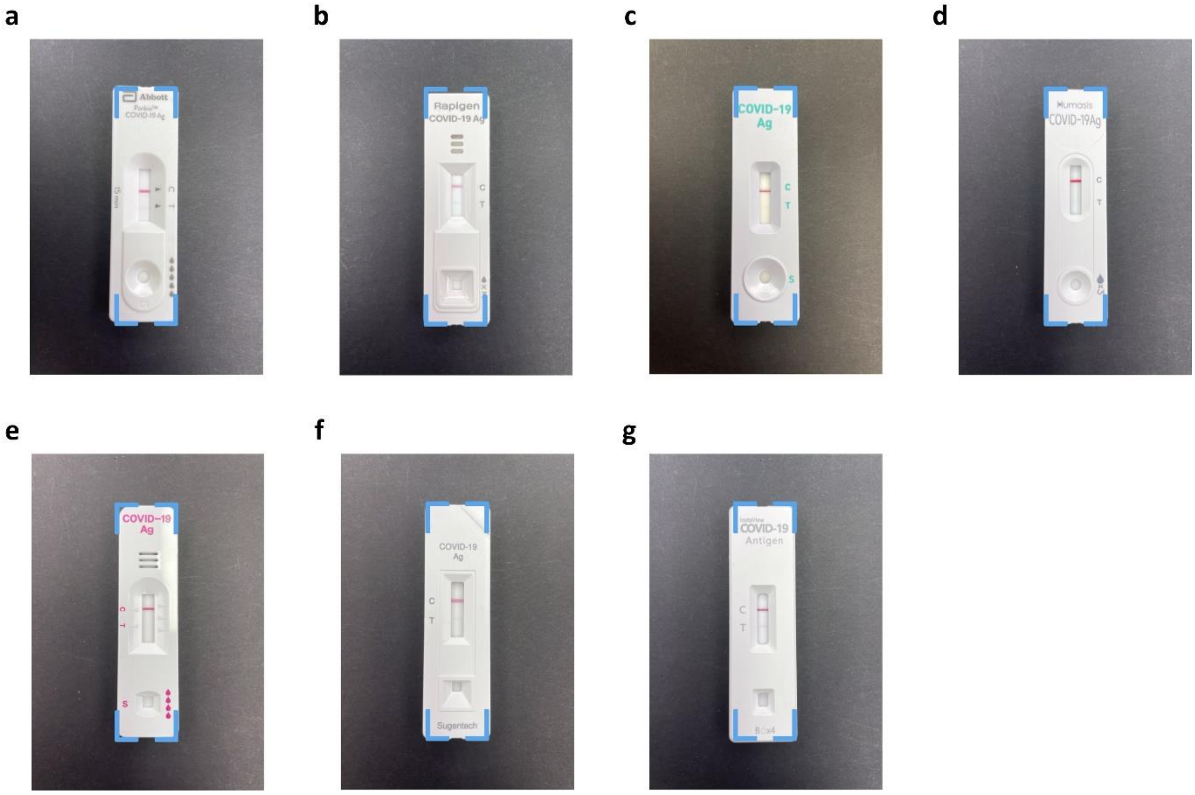


**Supplementary Fig. 5. Images of tested other corporation kit.** (a) Panbio COVID-19 Ag (Abbott, USA). (b) BIOCREDIT COVID-19 Ag (Rapigen, Republic of Korea). (c) SGTi-flex COVID-19 Ag (Sugentech, Republic of Korea). (d) GENEDIA COVID-19 (GCMS, Republic of Korea). (e) COVID-19 Ag Test (Humasis, Republic of Korea). (f) COVID-19 Ag (GenBody, Republic of Korea). (g) InstaView COVID-19 (SGmedical, Republic of Korea).

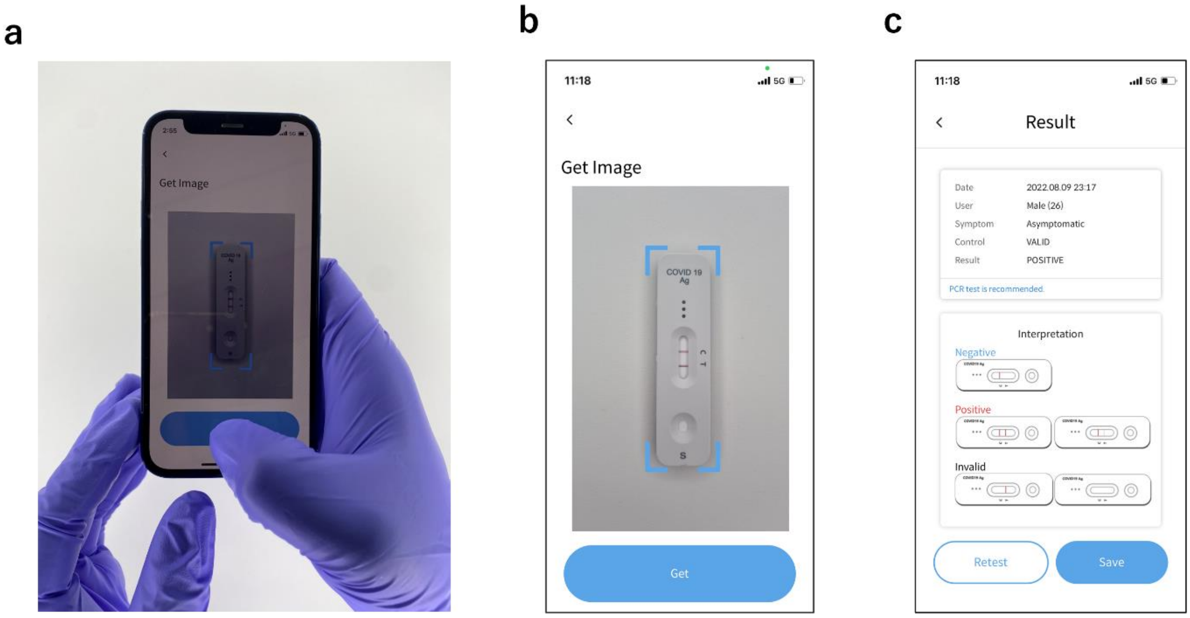


**Supplementary Fig. 6. Smartphone applications (apps).** (a-b) Apps for android phones and iPhones. Clinical images can be acquired using the App with the blue guidelines. (c) Smartphone-based artificial intelligence (AI) helps end users determine the clinical results (positive/negative/invalid).

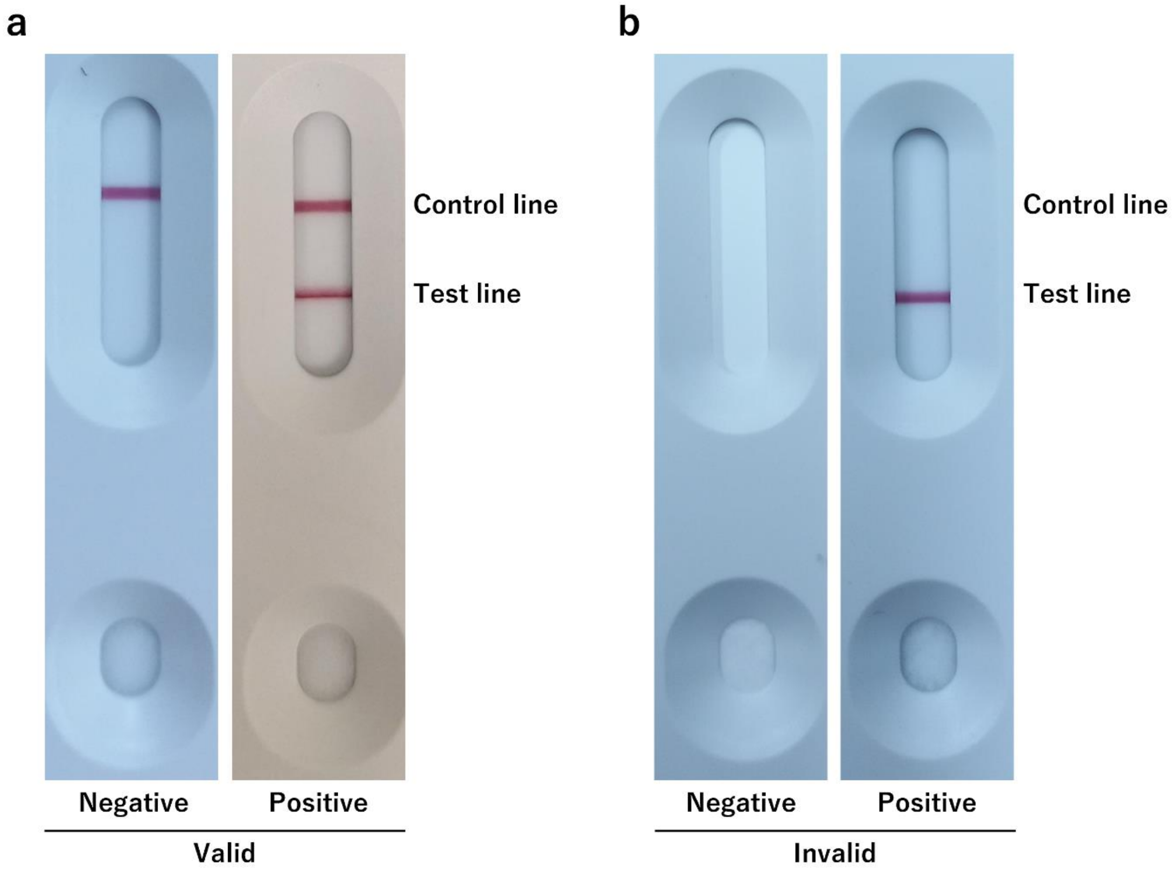


**Supplementary Fig. 7. Images of the valid and invalid cases in LFA test.** (a) Images of valid case; control line shows clear red line regardless of the sample lines. (b) Images of invalid case; control line shows no red line regardless of the sample lines.

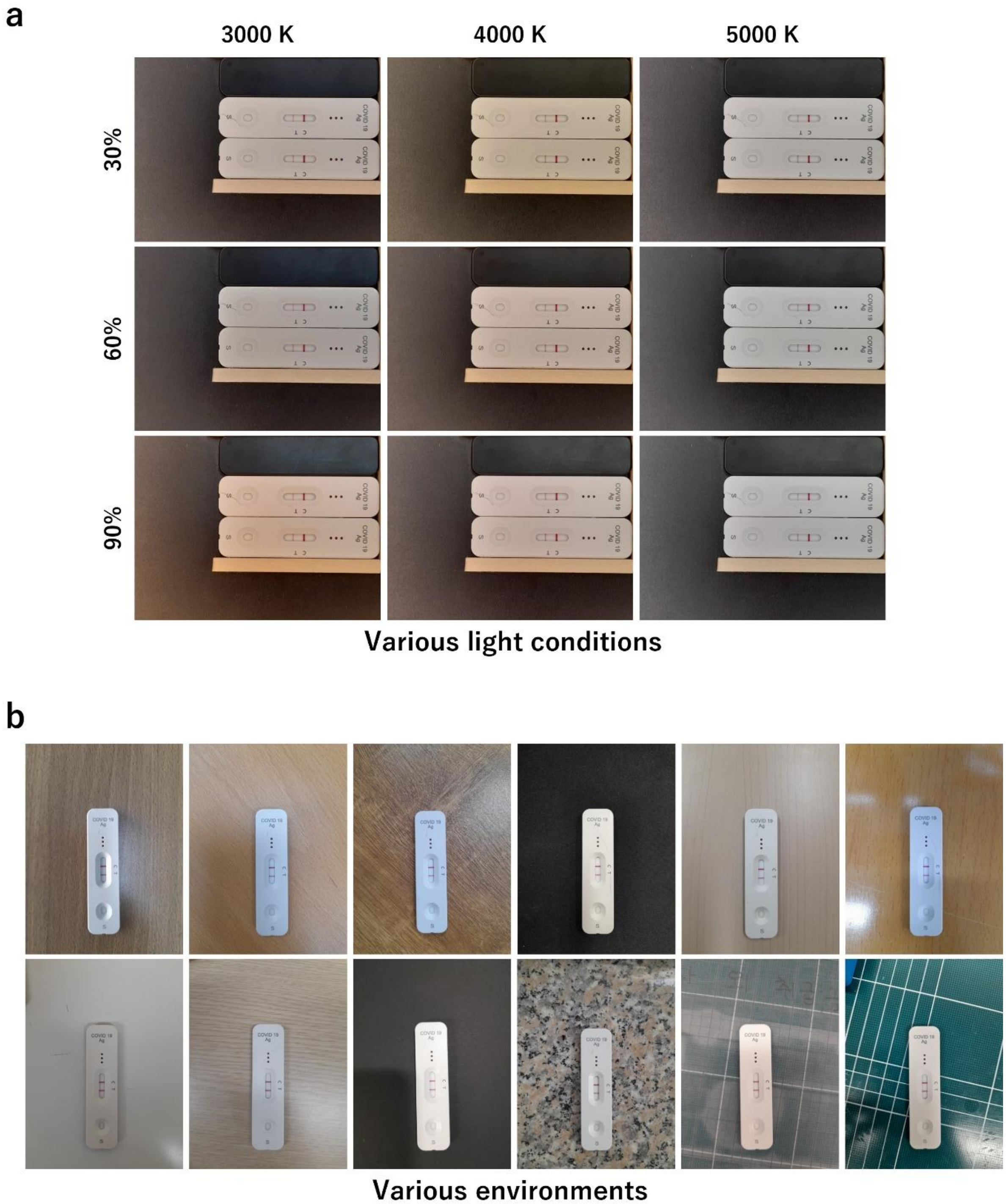


**Supplementary Fig. 8. LFA images of various surroundings.** Training and testing images under (a) various surroundings (brightness:0, 30, 60, 90% and color temperature: 3000, 4000, and 5000 K) and (b) indoor/outdoor, day/night, and various backgrounds.

**Supplementary Table 1.** Training datasets of standard and patient samples for algorithm development.

| Train set | Negative | Positive | Total |
|---|---|---|---|
| **Standard data** | 3,113 | 5,801 | 8,914 |
| **Patient data** | 2,338 | 5,667 | 8,005 |
| **Total** | 5,451 | 11,468 | 16,919 |

**Supplementary Table 2.** Test and validation datasets of patient samples for testing and evaluating the algorithm. We carried out all the clinical tests using patient samples in test and validation.

| Test & valid set | Patient data |
|---|---|
| **Healthy controls** | 432 |
| **Patients** | 1,026 |
| **Total** | 1,458 |

**Supplementary Table 3.** SARS-CoV-2 patients (n=45) and healthy controls (n=20) information including sample collection, variants, sex, ages, and Ct values.

| Patient number | Sample collections | Variants | Sex (0: female 1: male) | Ages | Ct values |
|---|---|---|---|---|---|
| **P#1** | Saliva | Omicron | 1 | 44 | 22.2 |
| **P#2** | Saliva | Omicron | 0 | 53 | 20.65 |
| **P#3** | Saliva | Omicron | 0 | 73 | 28.12 |
| **P#4** | Saliva | Omicron | 1 | 64 | 28.25 |
| **P#5** | Saliva | Omicron | 1 | 38 | 26.84 |
| **P#6** | Saliva | Omicron | 0 | 33 | 22.84 |
| **P#7** | Saliva | Omicron | 1 | 53 | 22.52 |
| **P#8** | Saliva | Omicron | 0 | 57 | 31.72 |
| **P#9** | Saliva | Omicron | 1 | 48 | 31.22 |
| **P#10** | Saliva | Omicron | 0 | 62 | 30.65 |
| **P#11** | Saliva | Omicron | 0 | 25 | 22.22 |
| **P#12** | Saliva | Omicron | 0 | 53 | 26.84 |
| **P#13** | NP/OP | Omicron | - | - | 21.44 |
| **P#14** | NP/OP | Omicron | - | - | 17.8 |
| **P#15** | NP/OP | Omicron | 1 | 44 | 18.72 |
| **P#16** | NP/OP | Omicron | 1 | 83 | 23.48 |
| **P#17** | NP/OP | Omicron | 0 | 33 | 22.84 |
| **P#18** | NP/OP | Omicron | 1 | 65 | 20.94 |
| **P#19** | NP/OP | Omicron | 0 | 39 | 22.7 |
| **P#20** | NP/OP | Omicron | 1 | 65 | 17.81 |
| **P#21** | NP/OP | Omicron | 0 | 25 | 22.22 |
| **P#22** | NP/OP | Omicron | 0 | 62 | 17.8 |
| **P#23** | NP/OP | Omicron | 0 | 53 | 26.84 |
| **P#24** | NP/OP | Omicron | 1 | 46 | 26.78 |
| **P#25** | NP/OP | Omicron | 0 | 78 | 28.12 |
| **P#26** | NP/OP | Omicron | 1 | 58 | 26.5 |

| | | | | | |
|---|---|---|---|---|---|
| **P#27** | NP/OP | Omicron | 1 | 46 | 25.69 |
| **P#28** | NP/OP | Omicron | 1 | 38 | 26.84 |
| **P#29** | NP/OP | Omicron | 1 | 64 | 28.25 |
| **P#30** | NP/OP | Omicron | 1 | 55 | 23.9 |
| **P#31** | NP/OP | Omicron | 1 | 22 | 26.78 |
| **P#32** | NP/OP | Omicron | 1 | 41 | 22.32 |
| **P#33** | NP/OP | Omicron | 1 | 41 | 22.32 |
| **P#34** | NP/OP | Omicron | 0 | 36 | 22.84 |
| **P#35** | NP/OP | Delta | 1 | 16 | 32 |
| **P#36** | NP/OP | Delta | 1 | 18 | 31 |
| **P#37** | NP/OP | Delta | 1 | 19 | 29 |
| **P#38** | NP/OP | Delta | 1 | 18 | 29 |
| **P#39** | NP/OP | Delta | 0 | 19 | 28 |
| **P#40** | NP/OP | Delta | 0 | 35 | 23.7 |
| **P#41** | NP/OP | Delta | 0 | 42 | 20.9 |
| **P#42** | NP/OP | Delta | 0 | 55 | 19.2 |
| **P#43** | NP/OP | Delta | 1 | 59 | 24.9 |
| **P#44** | NP/OP | Delta | 0 | 63 | 24.1 |
| **P#45** | NP/OP | Omicron | 1 | 27 | Daily progress |

| **healthy control** | **Sample type** | **Variant** | **Sex (0: female 1: male)** | **Ages** | **Ct value** |
|---|---|---|---|---|---|
| **N#1** | NP/OP | - | 1 | 34 | N |
| **N#2** | NP/OP | - | 1 | 26 | N |
| **N#3** | NP/OP | - | 1 | 27 | N |
| **N#4** | NP/OP | - | 1 | 38 | N |
| **N#5** | NP/OP | - | 1 | 27 | N |
| **N#6** | NP/OP | - | 1 | - | N |
| **N#7** | NP/OP | - | 1 | 27 | N |
| **N#8** | NP/OP | - | 1 | 34 | N |

| | | | | | |
|---|---|---|---|---|---|
| **N#9** | NP/OP | - | 0 | 31 | N |
| **N#10** | NP/OP | - | 0 | 34 | N |
| **N#11** | NP/OP | - | 1 | 27 | N |
| **N#12** | NP/OP | - | 1 | 26 | N |
| **N#13** | NP/OP | - | 0 | 23 | N |
| **N#14** | NP/OP | - | 1 | 23 | N |
| **N#15** | NP/OP | - | 1 | 26 | N |
| **N#16** | NP/OP | - | 0 | - | N |
| **N#17** | NP/OP | - | 1 | 26 | N |
| **N#18** | NP/OP | - | 1 | 27 | N |
| **N#19** | NP/OP | - | 0 | - | N |
| **N#20** | NP/OP | - | 1 | 27 | N |

**Supplementary Table 4.** Multi-users and multi-smartphone models for validating universality for the tests of six patients and three healthy controls (normal).

| User number | Smartphone model | Sex | Ages |
|---|---|---|---|
| **User 1** | LG Q51 | Female | 25 |
| **User 2** | Galaxy A52 | Male | 27 |
| **User 3** | iPhone 12 mini | Male | 37 |
| **User 4** | iPhone 11 Pro | Male | 25 |
| **User 5** | iPhone 14 Max | Male | 27 |

| | Ct values | Sex (0: female 1: male) | Ages |
|---|---|---|---|
| **P#1** | 18.7 | 1 | 44 |
| **P#2** | 22.2 | 0 | 25 |
| **P#3** | 22.84 | 0 | 33 |
| **P#4** | 26.78 | 1 | 22 |
| **P#5** | 26.84 | 1 | 38 |
| **P#6** | 31 | 1 | 18 |
| **N#1** | N | 0 | 31 |
| **N#2** | N | 1 | 23 |
| **N#3** | N | 0 | 55 |

**Supplementary Table 5.** Universality of different LFA kits. Validation with the LFAs of three different manufacturers and test kits from seven different manufacturers.

| Product (Corp.) for validation data | Negative | Positive | Total |
|---|---|---|---|
| **BIO CREDIT COVID-19 Ag (Rapigen)** | 60 | 60 | 120 |
| **COVID-19 Ag Home Test (SDbiosensor)** | 60 | 60 | 120 |
| **COVID-19 Ag Self Test (Yuhan)** | 60 | 60 | 120 |
| | 180 | 180 | 360 |

| Product (Corp.) for test data | Negative | Positive | Total |
|---|---|---|---|
| **Panbio COVID-19 Ag (Abbott)** | 450 | 900 | 1,350 |
| **BIO CREDIT COVID-19 Ag (Rapigen)** | 450 | 900 | 1,350 |
| **SGT-flex COVID-19 Ag (Sugentech)** | 450 | 900 | 1,350 |
| **GENEDIA COVID-19 (GCMS)** | 450 | 900 | 1,350 |
| **COVID-19 Ag Test (Humasis)** | 450 | 900 | 1,350 |
| **COVID-19 Ag (Genbody)** | 450 | 900 | 1,350 |
| **InstaView COVID-19 (SGmedical)** | 450 | 900 | 1,350 |
| **AllCheck COVID19 Ag (Calth)** | 432 | 1026 | 1458 |
| | 3,582 | 7,326 | 10,908 |
