# Supplementary material for: Sample-to-answer platform for the clinical evaluation of COVID-19 using a deep learning-assisted smartphone-based assay
Source: Nat Commun. 2023 Apr 24;14:2361. doi: 10.1038/s41467-023-38104-5 (PMC10124933; doi:10.1038/s41467-023-38104-5)
Supplement: Supplementary file 3 — Description of Additional Supplementary Information [file 41467_2023_38104_MOESM3_ESM.docx]

**Supplementary movie 1**

The demonstration of developed smartphone apps using the algorithm (SMART^AI^-LFA).

**Supplementary movie 2**

The demonstration of multi-user tests under various surroundings (such as indoors/outdoors, lighting conditions, and shade/sunlight with various backgrounds) with different smartphone models (LG Q51, Galaxy A52, iPhone 12 mini, iPhone 11 Pro, and iPhone 14 Max).

**Supplementary movie 3**

The demonstration of the universality by testing an additional seven different LFA models (Panbio COVID-19 Ag, BIO CREDIT COVID-19 Ag, SGT-flex COVID-19 Ag, GENEDIA COVID-19, Humasis COVID-19 Ag Test, Genbody COVID-19 Ag, InstaView COVID-19).
